# Albumin versus saline infusion for sepsis-related peripheral tissue hypoperfusion: a proof-of-concept prospective study

**DOI:** 10.1186/s13054-024-04827-0

**Published:** 2024-02-07

**Authors:** Paul Gabarre, Cyrielle Desnos, Alexandra Morin, Louai Missri, Tomas Urbina, Vincent Bonny, Matthieu Turpin, Jean-Luc Baudel, Laurence Berard, Melissa Montil, Bertrand Guidet, Guillaume Voiriot, Jérémie Joffre, Eric Maury, Hafid Ait-Oufella

**Affiliations:** 1 Medical Intensive Care Unit, Saint-Antoine University Hospital, APHP, Sorbonne University, 75012 Paris, France; 2 Intensive Care Unit, Tenon University Hospital, APHP, Sorbonne University, Paris, France; 3 Department of Pharmacology, Assistance Publique-Hôpitaux de Paris, Hôpital St Antoine, Paris, France; 4 Clinical Research Platform of East of Paris (URCEST-CRCEST-CRB), Sorbonne Université, Paris, France; 5 INSERM UMRS 938, Centre de Recherche Saint-Antoine, CRSA, Immune System and Neuroinflammation Laboratory, Hôpital Saint-Antoine, Sorbonne Université, 184 Rue du Faubourg Saint-Antoine, 75012 Paris, France; 6 Paris Cardiovascular Research Center, Inserm U970, University Paris Cité, Paris, France

**Keywords:** Sepsis, Albumin, Capillary refill time, Mottling, Tissue perfusion

## Abstract

**Background:**

Albumin has potential endothelial protective effects through antioxidant and anti-inflammatory properties. However, the effect of albumin on peripheral tissue perfusion in human sepsis remains poorly known.

**Methods:**

Bi-centric prospective study included patients with sepsis with or without shock and prolonged CRT > 3 s despite initial resuscitation. Clinicians in charge of the patients were free to infuse either saline 500 mL or human serum albumin 20% 100 mL over 15 min. Global hemodynamic parameters as well as peripheral tissue perfusion were analyzed after 1 (H1) and 4 h (H4). The primary endpoint was CRT normalization (< 3 s) at H1.

**Results:**

62 patients were screened, and 50 patients (13 sepsis and 37 septic shock) were included, 21 in the saline group and 29 in the albumin group. SOFA score was 8 [5–11], and SAPS II was 53 [45–70]. Median age was 68 [60–76] years with a higher proportion of men (74%). The primary sources of infection were respiratory (54%) and abdominal (24%). At baseline, comorbidities, clinical and biological characteristics were similar between groups**.** At H1, CRT normalization (< 3 s) was more frequent in patients receiving albumin as compared to patients treated by saline (63 vs 29%, *P* = 0.02). The decrease in fingertip CRT was more important in the albumin group when compared to saline group (− 1.0 [− 0.3; − 1.5] vs − 0.2 [− 0.1; − 1.1] seconds, *P* = 0.04) as well as decrease in mottling score. At H4, beneficial effects of albumin on peripheral tissue perfusion were maintained and urinary output trended to be higher in the albumin group (1.1 [0.5–1.8] vs 0.7 [0.5–0.9] ml/kg/h, *P* = 0.08). Finally, arterial lactate level did not significantly change between H0 and H4 in the saline group but significantly decreased in the albumin group (*P* = 0.03).

**Conclusion:**

In patients with resuscitated sepsis, albumin infusion might lead to greater improvement of tissue hypoperfusion compared to saline. ClinicalTrials.gov Identifier: NCT05094856.

**Supplementary Information:**

The online version contains supplementary material available at 10.1186/s13054-024-04827-0.

## Introduction

Sepsis is a common life-threatening condition in response to microbial injury, leading to tissue hypoperfusion, multiorgan failure and potentially to death. Despite some improvement in the early identification and management, sepsis remains a major issue worldwide responsible for unacceptable morbidity and mortality [1]. Nevertheless, thanks to clinical and experimental studies, the understanding of sepsis pathophysiology is improving. Recently, the contribution of the endothelium in severe infection-related tissue damage has been highlighted [2]. Briefly, during sepsis, endothelial responses are dysregulated and the glycocalyx layer is damaged with functional consequences including vasomotor tone dysregulation, activation of coagulation and ultimately decreased microcirculatory blood flow. Impaired microvascular blood flow evaluated either with sublingual videomicroscopy [3] or bedside clinical tools such as capillary refill time (CRT) [4] has been identified as a key pejorative factor in sepsis patients associated with organ failure severity as well as mortality.

Until now, there is no specific available treatment to limit endothelial dysfunction and consecutive decreased microvascular blood flow. Despite no clear benefit of human serum albumin on mortality in non-selected patients with septic shock [5], albumin still represents a potential treatment in sepsis [6]. Experimental studies have described protective functions of albumin in animal models of sepsis through oncotic, antioxidant and anti-inflammatory mechanisms [6]. Our group has reported, using acetylcholine iontophoresis coupled to laser Doppler, that albumin infusion strongly improved endothelial function in septic patients, whereas saline did not [7]. Whether such albumin-induced improved endothelial function may translate into better clinical tissue perfusion has not been studied.

This prospective study aimed to compare the impact of albumin *versus* saline infusion on peripheral tissue perfusion in a selected population of patients with sepsis and persistent impaired tissue hypoperfusion despite initial resuscitation.

## Methods

### Study design

We conducted a prospective study in 2 tertiary teaching hospitals (Saint-Antoine and Tenon hospitals, Paris, France), “Effects of Fluid Therapy on Peripheral Tissue Perfusion During Sepsis/Septic Shock” ClinicalTrials.gov Identifier: *NCT05094856* to compare the impact of albumin *versus* saline infusion on selected sepsis patients with persistent impaired tissue perfusion despite initial resuscitation. The trial was funded by Grifols which had no role in the conduct of the study, the reporting of the data or the supply of study fluids. Albumin administered during the study was provided by each participating institution as part of the clinical treatment of critically ill patients.

### Patients

Resuscitated sepsis patients (Third International Consensus Definitions [8]), older than 18 years, to whom the attending intensivist decided to administer an additional volume expansion were screened. Between H6 and H48 after ICU admission, patients with a prolonged fingertip CRT (> 3 s) despite initial fluid expansion (crystalloids 20 ml/Kg), infection source control and antibiotic administration, were included. Septic patients requiring vasopressors were included after hemodynamic stabilization defined by MAP > 65 mm Hg with no change in vasopressor dosage during the last 2 h. Clinicians in charge of the patients were free to infuse either saline 0.9% or albumin (Human Albumin Solution 20%).

Exclusion criteria for this trial were: COVID-19 disease, pregnancy, patient under Guardianship/Curatorship, opposition to participate, CRT not evaluable (dark or damaged skin), moribund patient, estimated life expectancy less than one month, no affiliation to a social security regimen.

### Treatment

Fluid therapy was started at H0. Volume expansion was standardized with the infusion of 500 mL of saline 0.9% or 100 mL HAS 20% over 15 min. Choosing HAS 20% over HAS 4–5% was preferred to minimize the administered fluid volume. After 1 h, if the clinician in charge of the patient decided on an additional fluid expansion, the same fluid as the one infused at H0 was used. The decision to repeat fluid infusion between H1 and H4 was solely determined by the attending physician, relying on CRT but also on other global hemodynamic and tissue perfusion parameters.

### Outcome

The primary endpoint was fingertip CRT normalization defined as CRT < 3 s at one hour (H1) after fluid challenge. Secondary endpoints included changes in both CRT (in seconds) and mottling score between H0 and H1, urinary output between H0 and H4 and variations of arterial lactate levels between H0 and H4.

### Patient management and data collection

Patients were admitted directly from the emergency department or medical wards. Circulatory support was guided by our local protocol, adapted from international guidelines [9]. Initial therapeutic management included antibiotic administration, fluid infusion (Crystalloids 20 mL/Kg), norepinephrine infusion to maintain a mean arterial pressure (MAP) > 65 mmHg and infection source control when available. All patients were investigated with transthoracic echocardiography (Vivid 7 Dimension’06, GE Healthcare) to assess left ventricular function, volume status and cardiac output. Repetitive transthoracic echocardiography was performed routinely during acute circulatory failure management. General characteristics of the patients were recorded: demographic data, diagnoses, severity of illness evaluated by the Sequential Organ Failure Assessment (SOFA) score [10] and Simplified Acute Physiology Score II (SAPS II) [11]. We collected MAP, heart rate (HR) and cardiac index. Tissue and organ perfusion were assessed through arterial lactate level, urinary output, mottling score and fingertip CRT.

The same physician did CRT measurements at H0, H1 and H4 to limit inter-rater variability. As previously reported and standardized by our group, CRT was measured by applying firm pressure to the distal phalanx of the index finger for 15 s. The pressure applied was just enough to remove the blood at the tip of the physician’s nail, illustrated by the appearance of a thin white distal crescent (blanching) under the nail. A chronometer recorded the time for the return to the baseline color. CRT was measured twice, and the mean value was recorded. [12]

### Statistics

In a preliminary study including 25 patients with persistent prolonged CRT after initial resuscitation, we found that saline infusion induced CRT normalization at 1 h in only 4 patients (16%). Assuming that albumin increases the proportion of CRT normalization from 16 to 40%, the trial was designed to enroll 60 patients in order to provide a power of 90% with an alpha level of 5%. Continuous variables were presented as median and 25th–75th interquartile ranges (IQR). Discrete variables were presented as percentages. Comparisons between groups were made with Fisher test for discrete variables and Mann–Whitney U test for continuous variables. Comparisons between before and after fluid infusion were made using a paired Wilcoxon signed-rank test. Statistical analysis and graphical representations were performed using GraphPad Prism 10.2 software (Graph Pad Software Inc., La Jolla, CA). A two-sided *P* value of less than 0.05 was considered statistically significant.

## Results

Between February 2022 and February 2023, 62 patients with sepsis were initially included and the study was stopped because the estimated patient number to be included was reached. Twelve patients were finally excluded leaving 50 patients (13 sepsis and 37 septic shock) for analysis (Additional file 1: Figure S1). Median age was 68 [60–76] years with a higher proportion of men (74%). The main sources of infection were respiratory (54%) and abdominal (24%). Included patients had severe disease with high SOFA scores (8 [5–11]) and high SAPS II (53 [45–70]). Organ support therapy is detailed in Table 1. In-ICU mortality was 34% (17/50). Before inclusion, all patients were resuscitated and received antibiotics and crystalloids. At inclusion, patients received fluid expansion alternatively with either saline (N = 21) or albumin (N = 29). Comorbidities, clinical and biological characteristics were similar between groups **(**Table 1**)**. The proportion of septic shock was similar between groups (62% vs 69%, *P* = 0.59). Baseline norepinephrine dosage trended to be higher in the albumin group. Baseline fingertip CRT, mottling score and arterial lactate levels were not different between saline and albumin groups.

**Table 1:** Characteristics of included patients

Parameters	Total (n = 50)	Saline (n = 21)	Albumin (n = 29)	P value
General characteristics				
Age, years	68 [60–76]	68 [62–74]	70 [78–85]	0.83
BMI, kg/m^2^	23.5 [21.4–28.3]	25.2 [22.8–29.1]	22.9 [18.9–26.9]	0.06
Female	13 (26)	3 (14)	10 (34)	0.11
Comorbidities				
Vascular disease	16 (32)	9 (43)	7 (24)	0.22
Hypertension	21 (42)	10 (48)	11(38)	0.57
Diabetes	10 (20)	4 (19)	6 (21)	> 0.99
Chronic kidney disease	4 (16)	2 (10)	5 (17)	0.68
Cancer	27 (54)	13 (62)	14 (48)	0.40
Primary site of infection				
Lung	27 (54)	14 (67)	13 (45)	0.16
Abdomen	12 (24)	5 (24)	7 (24)	> 0.99
Urinary	9 (18)	2 (9)	7 (28)	0.27
Soft tissue	2 (4)	0 (0)	2 (7)	0.22
Time before inclusion, hour	12 [6–15]	12 [6–20]	8 [6–14]	0.10
Fluid resuscitation before inclusion, L	2 [1.5–2.5]	1.5 [1.4–2.5]	2 [1.5–2.5]	0.53
Severity score				
SAPS II	53 [45–70]	52 [45–66]	55 [43–73]	0.75
SOFA	8 [5–11]	8 [6–11]	8 [5–11]	0.75
Organ support therapy				
Norepinephrine				
N	33 (66)	13 (62)	20 (69)	0.59
Dose, µg/kg/min	0.5 [0.3–0.9]	0.4 [0.3–0.5]	0.7 [0.3–1.0]	0.06
Invasive mechanical ventilation	28 (56)	13 (62)	15 (52)	0.57
Global hemodynamics				
Heart rate, bpm	98 [77–115]	91 [77–115]	101 [85–115]	0.60
Mean arterial pressure, mmHg	72 [67–81]	75 [67–78]	72 [69–81]	0.93
Echocardiography				
Cardiac index, L/min/m^2^	2.3 [2–2.9]	2.4 [2.2–2.9]	2.3 [1.9–2.8]	0.56
LVEF, %	50 [45–60]	50 [49–55]	50 [45–60]	0.54
Tissue perfusion				
Knee CRT	4.4 [3.5–5.3]	4.3 [3.6–5]	4.6 [3.5–5.3]	0.78
Index CRT	3.5 [3.2–3.9]	3.5 [3.2–3.9]	3.6 [3.4–4.0]	0.47
Mottling score	1 [1, 2]	2 [1–3]	1 [1, 2]	0.34
Biology				
Arterial lactate, mmol/l	2.2 [1.3–3.1]	2.2 [1.3–3.1]	2.4 [1.2–3.2]	0.62
Creatininemia, µmol/l	112 [76–230]	117 [84–194]	106 [68–269]	0.94
Albuminemia, g/l	22.4 [20–25.7]	20.6 [16.6–22.2]	23.6 [21.1–26]	0.15

Data shown as median [25e−75e percentiles] for continuous data, number (percentage) for categorical data; SAPS II = Simplified Acute Physiology Score II; Data shown as median [25e−75e percentiles] for continuous data, number (percentage) for categorical data; and SAPS II = Simplified Acute Physiology Score II

### Analysis at H1

**Fig. 1:**
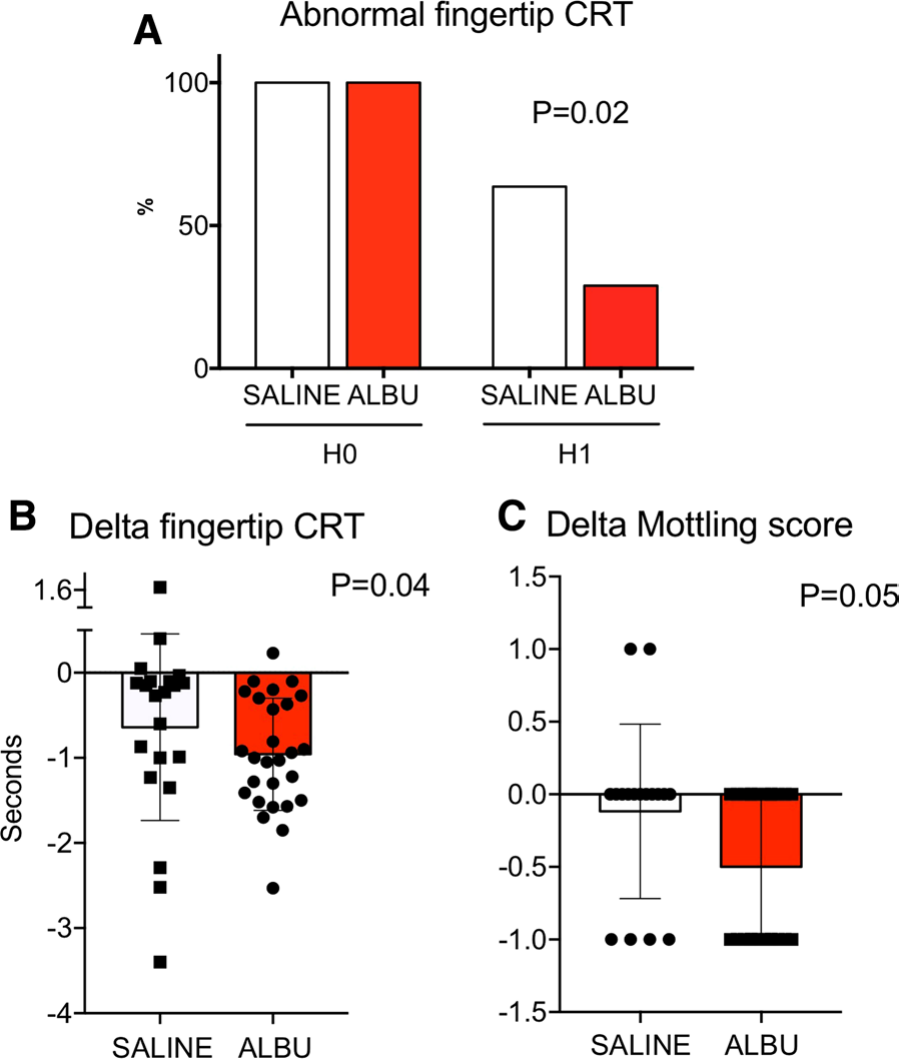
Evaluation of peripheral tissue perfusion at H1. **A** Percentage of patients with abnormal fingertip CRT, defined as > 3 s at baseline and 1 h after saline (white bars) or Albumin (red bars) infusion. **B** Variations of fingertip CRT between H0 and H1 (in seconds). **C** Variations of mottling score between H0 and H1. **A** Fisher test; **B**, **C** Nonparametric Mann–Whitney test

One hour after fluid expansion, we observed a slight but not significant decrease in norepinephrine dose in both groups. Heart rate, mean arterial pressure and cardiac index were not statistically different between H0 and H1 in either groups (Table 2). Interestingly, CRT normalization (< 3 s) at H1 was more frequently observed in patients receiving albumin compared to those treated with saline (63 vs 29%, *P* = 0.02) (Fig. 1A). Changes in CRT did not correlate with cardiac output variations (Additional file 2: Figure S2). In addition, we observed that the variation of fingertip CRT was larger in the albumin group when compared to saline group (− 1.0 (− 0.3; − 1.5) vs − 0.2 (− 0.1; − 1.1) seconds, *P* = 0.04) (Fig. 1B**)**. We also analyzed the impact of fluid therapy on mottling extension around the knee, another classical parameter of peripheral tissue perfusion. Therefore, we observed a more important decrease in mottling score in patients receiving albumin than in patients treated with saline (− 0.5 (0; −1) vs 0 (0; −0.5), *P* = 0.05) (Fig. 1C**)**.

**Table 2:** Hemodynamic parameters before (H0) and 1 h (H1) after fluid infusion

	Saline (n = 21)			Albumin (n = 29)			*P* value At H1
	H0	H1	*P* value *H0–H1	H0	H1	*P* value* H0–H1	*P* value Sal vs Alb #
HR, bpm	91 [77–115]	95 [71–122]	0.50	101 [85–115]	100 [82–116]	0.14	0.65
MAP, mmHg	75 [67–78]	74 [68–79]	> 0.99	72 [69–81]	76 [70–84]	0.27	0.33
Norepinephrine, n(%)	13 (62)	13 (62)	> 0.99	20 (69)	(68)	> 0.99	> 0.99
Dose, µg/kg/min	0.39 [0.3–0.48]	0.30 [0.29–0.4]	0.08	0.70 [0.3–1]	0.63 [0.3–0.9]	0.25	0.03
Cardiac index, L/min/m^2^	2.4 [2.2–2.9]	2.5 [2–2.8]	0.65	2.3 [1.9–2.8]	2.5 [2–2.9]	0.15	0.90
CRT (sec)	3.5 [3.2–3.9]	3.1 [2.6–3.4]	< .001	3.6 [3.4–4.0]	2.7 [2.1–3.2]	< .001	0.13
Mottling score	2 [1–3]	1 [1–3]	0.50	1 [1–3]	1 [0–2]	< .001	0.18

HR, heart rate; MAP, mean arterial pressure

*paired test; #non-paired test

### Analysis At H4

**Fig. 2:**
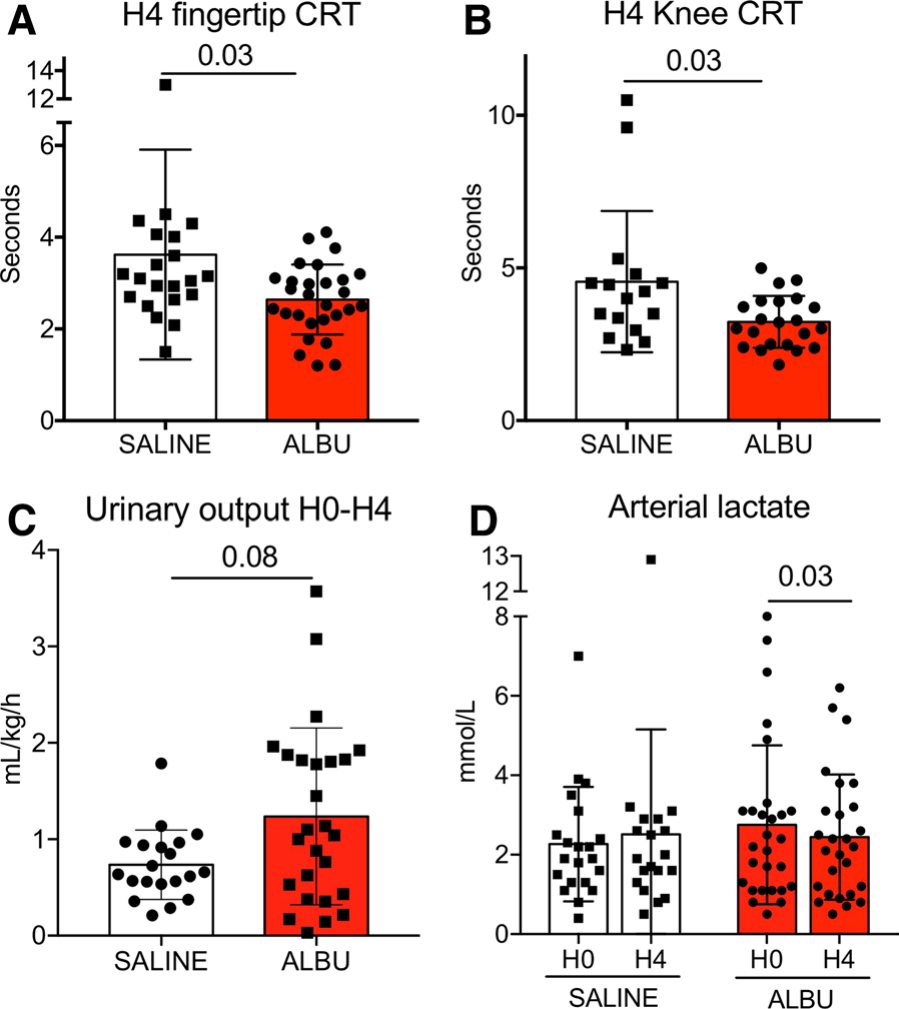
Evaluation of peripheral tissue perfusion at H4. **A** Fingertip CRT at H4 in patients treated by saline (white) or albumin (red). **B** Knee CRT at H4 in patients treated by saline (white) or albumin (red). **C** Urinary output between H0 and H4 in patients treated by saline (white) or albumin (red). **D** arterial lactate levels in patients treated by saline (white) or albumin (red) at H0 and H4 **A**–**C** nonparametric Mann–Whitney test and **D** paired Wilcoxon signed-rank test

Between H1 and H4, 25 patients received one additional fluid infusion, 10/22 (48%) in the saline group and 15/29 (52%) in the albumin group. At H4, heart rate, mean arterial pressure, norepinephrine dose and cardiac output were not statistically different between groups (Table 2). At H4, CRT was significantly lower in the albumin group on the fingertip (2.7 [2.3–3.1] vs 3.1 [2.7–4] seconds, *P* = 0.03) as well as on the knee area (3.2 [2.5–3.9] vs 4.1 [3.3–4.6] seconds, *P* = 0.03) (Fig. 2A, B). We analyzed urinary output and arterial lactate levels, two other parameters of organ perfusion. We found a trend for higher urinary output in the albumin group (1.1 [0.5–1.8] vs 0.7 [0.5–0.9] ml/kg/h, *P* = 0.08). Arterial lactate level did not significantly change between H0 and H4 in the saline group (Fig. 2C) but significantly decreased in the albumin group (Fig. 2D).

## Discussion

In this prospective study performed on resuscitated patients with sepsis and persistent tissue hypoperfusion, we showed that albumin infusion improved both peripheral and global tissue perfusion more than saline.

Here, we focused on patients with persistent tissue hypoperfusion despite initial resuscitation, a population representing less than 20% of patients with resuscitated sepsis in the emergency ward [13] but characterized by poor outcome [12, 13]. Patients were screened using the fingertip CRT, a validated bedside tool to identify the more severe patients [4] and to accurately monitor the effects of fluid challenge [14]. In addition, CRT is a safe and promising clinical tool to guide resuscitation in ICU patients as shown in the ANDROMEDA trial [15].

Here, we observed the probable beneficial effect of albumin on sepsis induced tissue hypoperfusion over saline. Beneficial effects are unlikely due to effects on macrohemodynamic since heart rate, MAP and cardiac output were not different between groups at baseline and after fluid challenge. In addition, changes in cardiac output did not correlate with CRT changes at H1 but we cannot definitively rule out that the beneficial effects of albumin on peripheral tissue perfusion were, at least in part, mediated by changes in cardiac index at early timepoints. Furthermore, the variability in cardiac index measurements using echocardiography may also be a confounder [16]. We speculated that albumin may act directly on the vascular wall, improving recovery of endothelium dysfunction and glycocalyx damage through multiple ways [6]. Due to its amphoteric nature, albumin promotes tight binding with the glycocalyx, while its negative charge participates in its parietal electrical barrier [17]. Next, albumin has well characterized antioxidant functions which is relevant in the context of sepsis, where high oxidative state participates in endothelial NO synthase dysregulation, leading to impaired vascular tone. Albumin has a free thiol group in reduced form carried by a cysteine residue, allowing deleterious plasma free radicals to be scavenged. Finally, albumin can complex with heavy metals, protecting them from oxidation by the Fenton reaction. In rodent models, albumin had immunomodulatory effects attenuating NF-kB pathway activation and both IFN-γ, TNF-α production. The decrease in the inflammatory response following albumin administration was also found in experimental hemorrhagic shock and was associated with an improvement in the mesenteric microcirculatory perfusion [18]. Measurements of glycocalyx or inflammatory biomarkers would be helpful to support our pathophysiological hypothesis on the protective effect of albumin on the vascular wall. Finally, difference between groups may also be due to deleterious effects of saline infusion. Indeed, experimental works suggest that saline impairs endothelial barrier and aggravates glycocalyx shedding [19].

In humans, some works supported the vascular protective effect of albumin. In a sub-study of ALBIOS trial including 375 patients with septic shock, it was reported that soluble level of VE-cadherin, reflecting endothelial shedding, decreased in patients receiving albumin [20]. Our group has also showed that albumin strongly improved acetylcholine-mediated endothelial reactivity in patients with sepsis, whereas saline had no effect [7]. We found beneficial effect of albumin on peripheral skin tissue perfusion but also on other organ perfusion as illustrated by higher urinary output and decreased lactate levels. Previous works from our group have shown that mottling score and CRT, 2 markers of skin perfusion, strongly correlated with urinary output and lactate levels. More recently, Huang et al. reported on a prospective cohort of critically ill patients that prolonged CRT was independently associated with sublingual microvascular flow abnormalities [21].

We acknowledge some limitations to this prospective study. Firstly, the constrained sample size might lack the power to discern potential differences between groups. Next, patients were not randomized and clinicians were not blinded to the infused treatment. The decision to administer saline or albumin, left to the discretion of the attending physician, may pose a confounding factor, and the specific rationale for their choice was not recorded. Finally, as delineated in the methods section, we conducted a “real-life” study wherein the decision to administer a fluid challenge was solely determined by the attending physician, relying on non-standardized hemodynamic parameters to align with customary clinical practice. Overall, our results need to be confirmed in a larger cohort with a randomized double blind protocol.

## Conclusion

This prospective study on patients with sepsis highlights that albumin infusion compared to saline might lead to greater improvement of tissue perfusion.

### Supplementary Information

**Additional file 1.** Flow chart.

**Additional file 2.** Correlation between variations of cardiac index (H1-H0) and variations of fingertip CRT (H1-H0).

## Data Availability

The data sets used and analyzed during the current study are available from the corresponding author upon reasonable request.
