# Supplementary material for: Albumin versus saline infusion for sepsis-related peripheral tissue hypoperfusion: a proof-of-concept prospective study
Source: Crit Care. 2024 Feb 7;28:43. doi: 10.1186/s13054-024-04827-0 (PMC10848485; doi:10.1186/s13054-024-04827-0)
Supplement: Supplementary file 1 — Additional file 1. Flow chart. [file 13054_2024_4827_MOESM1_ESM.pptx]

## Slide 1
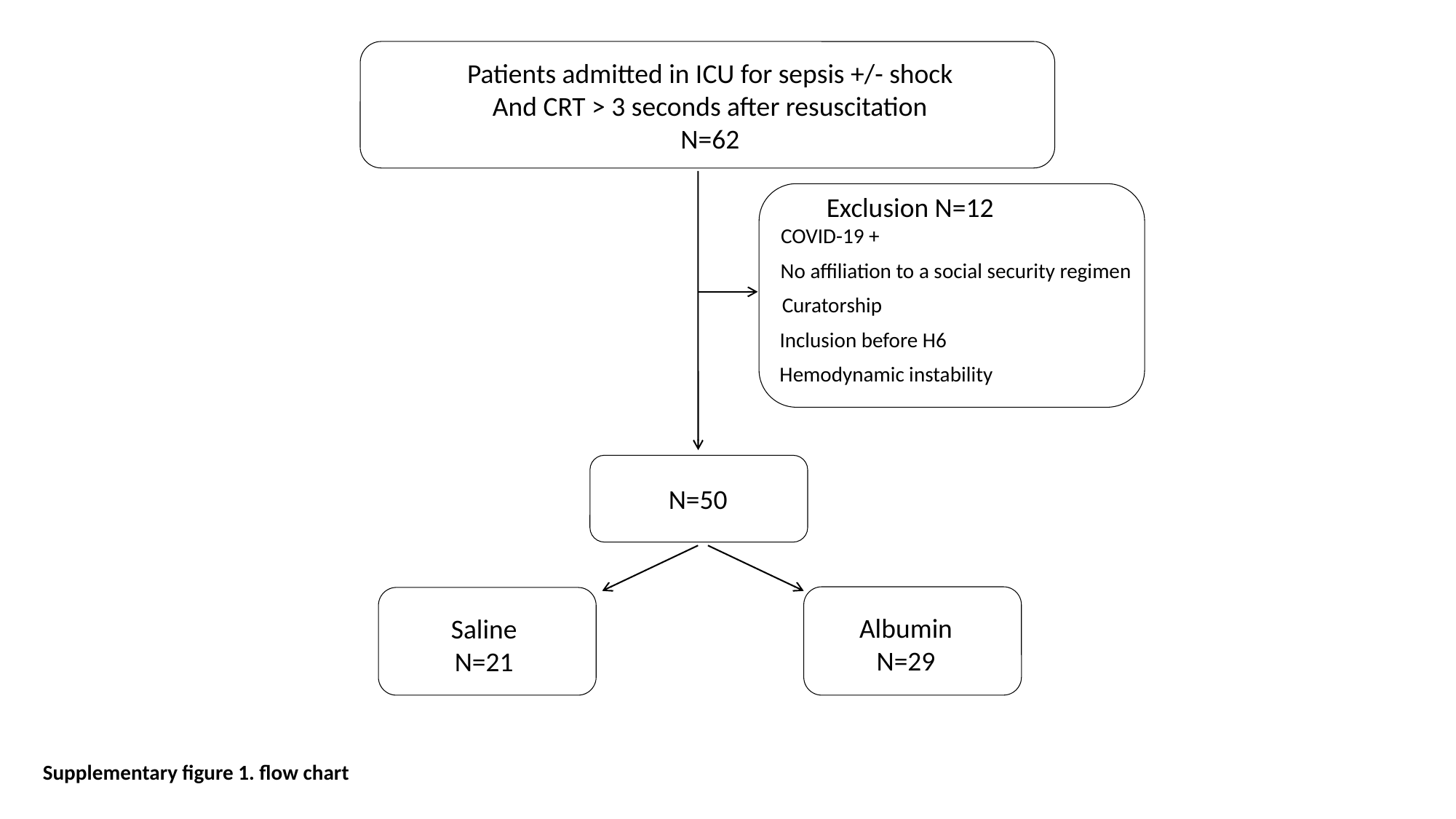

Patients admitted in ICU for sepsis +/- shock
And CRT > 3 seconds after resuscitation
N=62
Exclusion N=12
COVID-19 +
No affiliation to a social security regimen
Curatorship
Inclusion before H6
Hemodynamic instability
N=50
Albumin
N=29
Saline
N=21
Supplementary figure 1. flow chart

## Slide 2
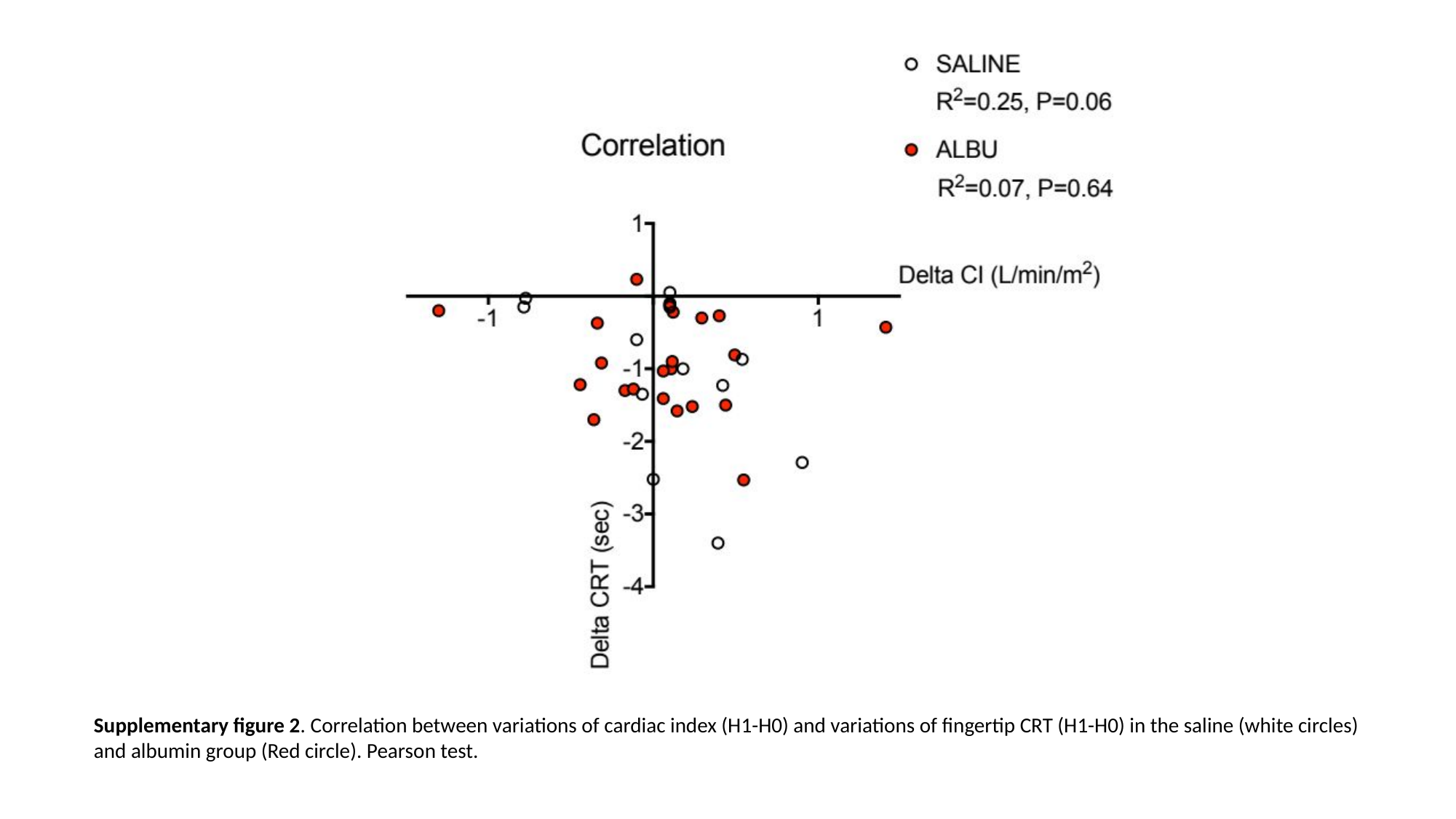

Supplementary figure 2. Correlation between variations of cardiac index (H1-H0) and variations of fingertip CRT (H1-H0) in the saline (white circles) and albumin group (Red circle). Pearson test.
