# Supplementary material for: Albumin versus saline infusion for sepsis-related peripheral tissue hypoperfusion: a proof-of-concept prospective study
Source: Crit Care. 2024 Feb 7;28:43. doi: 10.1186/s13054-024-04827-0 (PMC10848485; doi:10.1186/s13054-024-04827-0)
Supplement: Supplementary file 2 — Additional file 2. Correlation between variations of cardiac index (H1-H0) and variations of fingertip CRT (H1-H0). [file 13054_2024_4827_MOESM2_ESM.docx]

Supplemental Table 1

| **Parameters at H4** | **Saline**  **N=21** | **Albumin**  **N=29** | **P Value** |
| --- | --- | --- | --- |
| Heart rate, bpm | 96 [78-112] | 96 [80-113] | 0.87 |
| MAP, mmHg | 72 [68-77] | 74 [69-83] | 0.47 |
| Norepinephrine  N  Dose µg/kg/min | 16 (76)  0.38 [0.24-0.55] | 21 (72)  0.63 [0.19-1] | >0.99  0.76 |
| Cardiac index, mL/Kg/min | 2.4 [1.7-2.9] | 2.5 [2.0-2.8] | 0.60 |
| Finger tip CRT, sec | 3.1 [2.7-4] | 2.7 [2.3-3.1] | 0.03 |
| Knee CRT, sec | 4.1 [3.3-4.6] | 3.2 [2.5-3.9] | 0.03 |
| Mottling score | 1 [0-2] | 1 [0-1] | 0.18 |
| Fluid expansion H1-H4  N (%) | 10 (48) | 15 (52) | >0.99 |
